# Chromosome-scale assembly of the wild cereal relative *Elymus sibiricus*

**DOI:** 10.1038/s41597-024-03622-4

**Published:** 2024-07-26

**Authors:** Wenjie Shen, Bo Liu, Jialei Guo, Ying Yang, Xiaohui Li, Jie Chen, Quanwen Dou

**Affiliations:** 1 Key Laboratory of Adaptation and Evolution of Plateau Biota, Northwest Institute of Plateau Biology, Chinese Academy of Sciences, Xining, 810008 Qinghai China; 2 University of Chinese Academy of Sciences, Beijing, 101408 China; 3 Qinghai Provincial Key Laboratory of Crop Molecular Breeding, Northwest Institute of Plateau Biology, Chinese Academy of Sciences, Xining, 810008 Qinghai China

**Keywords:** Genome, Genomics

## Abstract

*Elymus* species, belonging to Triticeae tribe, is a tertiary gene pool for improvement of major cereal crops. *Elymus sibiricus*, a tetraploid with StH genome, is a typical species in the genus *Elymus*, which is widely utilized as a high-quality perennial forage grass in template regions. In this study, we report the construction of a chromosome-scale reference assembly of *E. sibiricus* line Gaomu No. 1 based on PacBio HiFi reads and chromosome conformation capture. Subgenome St and H were well phased by assisting with kmer and subgenome-specific repetitive sequence. The total assembly size was 6.929 Gb with a contig N50 of 49.518 Mb. In total, 89,800 protein-coding genes were predicted. The repetitive sequences accounted for 82.49% of the genome in *E. sibiricus*. Comparative genome analysis confirmed a major species-specific 4H/6H reciprocal translocation in *E. sibiricus*. The *E. sibiricus* assembly will be much helpful to exploit genetic resource of StH species in genus *Elymus*, and provides an important tool for *E. sibiricus* domestication.

## Background & Summary

The genus *Elymus* L. belongs to the grass tribe Triticeae, containing approximately 150 species^1,2^. The genus is entirely composed of polyploidy species with StH, StY, StHY, StPY, and StWY, including five basic genomes. The included basic genomes St, H, P, W are derived from *Pseudoroegeneria* (Neveski) Löve, *Hordeum* L., *Agropyron* Gaertn., and *Australopyrum* (Neveski) Love, respectively, although the origin of Y genome is still unknown^3,4^. *Elymus* species belong to the same tribe with staple food crops such as wheat (*Triticum aestivum*, 2n = 6x = 42; AABBDD genome), rye (*Secale cereal*, 2n = 2x = 14), and barley (*Hordeum vulgare*, 2n = 2x = 14), and which are important genetic resources with high diversity, constituting a tertiary gene pool for improvement of major cereal crops.

*Elymus sibiricus* L. (Siberian wild rye), a typical species of the genus *Elymus*, is a well-known perennial and caespitose grass. *E. sibiricus* is widely distributed in the northern hemisphere, with particular preponderance in Sweden, northern Asia, Japan, and North America^5^, which is mostly utilized as perennial forages in template regions^6,7^. *E. sibiricus* is an allotetraploid with a genome constitution of StStHH (2n = 4x = 28)^2^. Chromosomal polymorphisms and major rearrangements of *E. sibiricus* have been revealed by Florescence *in situ* hybridization (FISH) in different accessions^8,9^. Genomic SSR markers were exploited by screening enriched microsatellite DNA library for genetic diversity evaluation^10^. Transcriptome of *E. sibiricus* was profiled to reveal candidate genes connected to seed shattering^11^. Genome sequencing was carried out by Illumina HiSeq X-ten platform, and a draft genome of 4.34 Gb was assembled, and which was used for SSR markers development^12^.

**Fig. 1:**
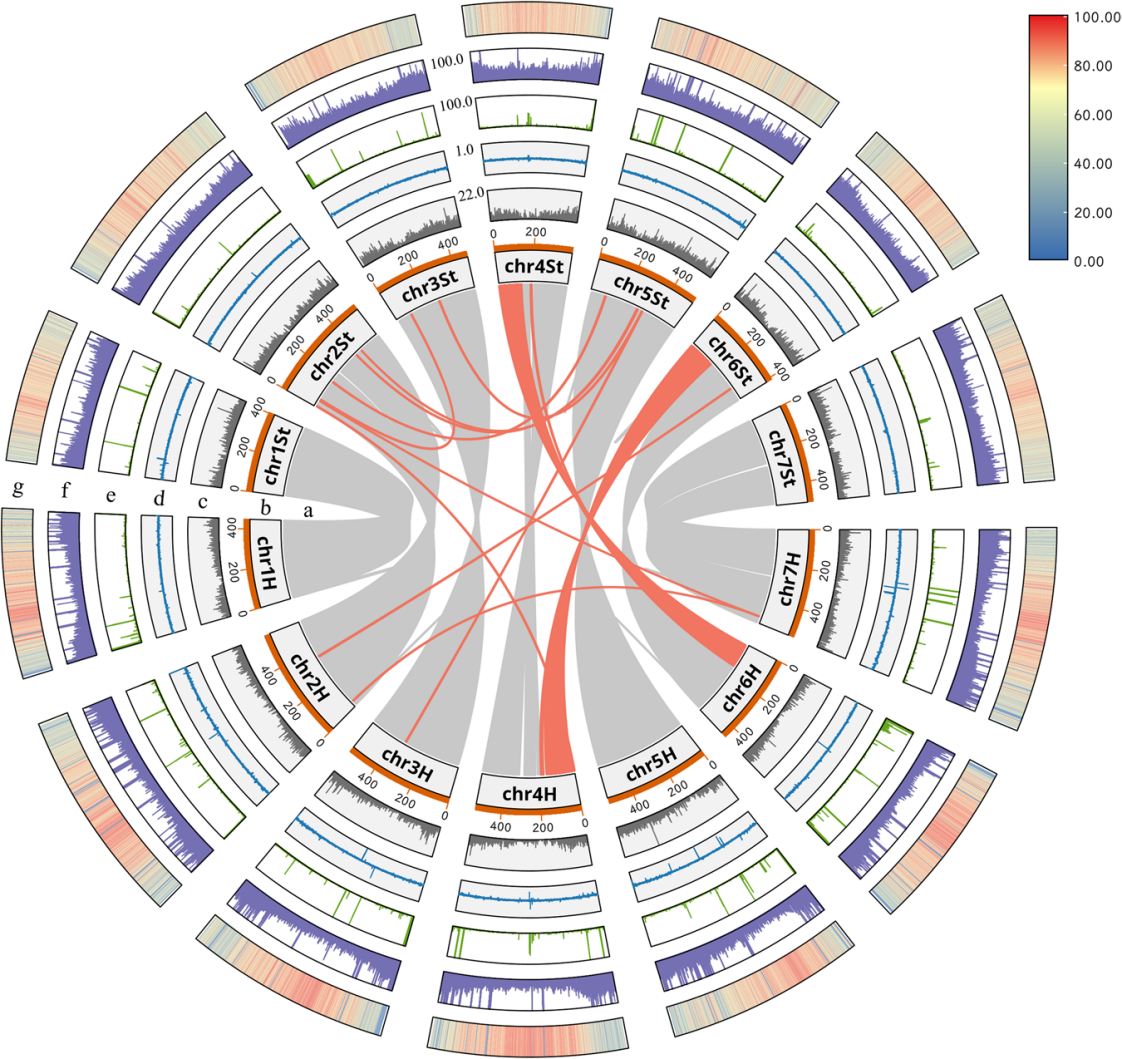
Overview of the assembled *E. sibiricus* genome. (**a**–**g**) are as followers: collinearity between the chromosomes (The color red is used to highlight the associations between different homologous), chromosomes, gene counts, GC content, Simple repeat density, DNA transposons density, LTR elements density (The window used for calculating the density of the above elements is 100,000 bp).

In this study, an *E. sibiricus* chromosome-scale reference genome by integrating PacBio HiFi reads and chromatin conformation capture data was assembled. The high-quality *E. sibiricus* assembly obtained in this study provides a reference for the StH genome of the genus *Elymus* in the Triticeae tribe (Fig. 1). It will be much helpful to facilitate genetic resource evaluation of StH species in genus *Elymus*. Furthermore, it can be served as important tool to directly domesticate *E. sibiricus* as a forage crop or even a cereal crop.

## Methods

### Plant materials and genome sequencing

The inbreed line Gaomu No.1 of *E. sibiricus* required for sequencing was self-crossed exactly 6 generations. Fresh young leaf tissue of it was collected, frozen in liquid nitrogen, The extraction of DNA samples follows the CATB method^13^. The DNA library preparation and sequencing were carried out according to the protocol provided in the SMRTbell® prep kit 3.0 instruction manual and sequencing was performed on the PacBio Revio platform. DNA required for Hi-C sequencing was purified using the QIAamp DNA Mini Kit (CAT#51306, Qiagen) following the manufacturer’s protocol, while for Next-Generation Sequencing (NGS) whole genome sequencing, libraries were constructed using the MGIEasy Universal DNA Library Prep Kit V1.0 (CAT#1000005250, MGI) following the standard protocol. The Hi-C library was sequenced on the DNBSEQ-T7 platform, while NGS for whole-genome sequencing was conducted on the MGISEQ-2000 platform. Fastp v0.23.4^14^ with default parameters was used to obtain NGS clean reads. All genome sequencing and Hi-C sequencing data were derived from a single plant. The data obtained from each platform is shown in Table 1.

**Table 1:** Data Output Statistics.

	Total reads	Total bases(bp)	Clean reads	Clean bases(bp)	Q20 rate (%)	Q30 rate (%)	GC (%)	Coverage
WGS	2,464,238,466	369,635,769,900	2,464,238,342	368,541,278,572	98.10	93.64	45.55	53.19x
HiC	5,068,172,198	760,225,829,700	5,068,171,148	759,706,246,428	98.50	94.77	46.38	109.64x
HiFi	—	—	12,131,005	210,308,786,786	95.19	88.50	45.04	30.35x

Raw reads from full-length transcriptome sequencing were processed into circular consensus (CCS) reads based on the adapter. Subsequently, full-length, non-chimeric (FLNC) transcripts were identified by detecting the poly A tail signal and 5′ and 3′ cDNA primers in CCS. Clustering was performed on full-length sequences from the same transcript, grouping similar full-length sequences into clusters, and obtaining a consensus sequence for each cluster. These consensus sequences were then corrected to obtain high-quality sequences for further analysis. High-quality FL transcripts from Iso-Seq were used to remove redundancy using cd-hit v4.8.1^15^ (identity >0.99).

### Genome assembly and chromosome construction

**Fig. 2:**
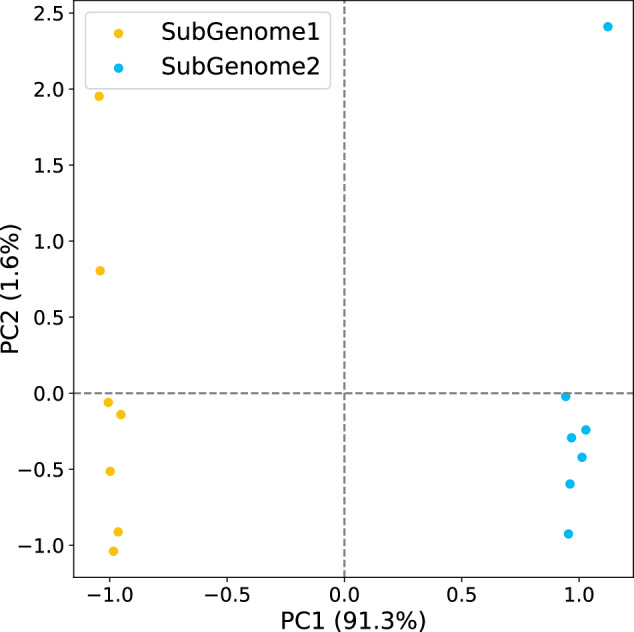
Principal component analysis (PCA) based on subgenome-specific kmers (kmer = 15).

The genome of *E. sibiricus* at the contig level was assembled using the hifiasm v0.19.6^16^, supplemented by Hi-C data and Pacbio HiFi data. Conserved homologous probes^17^ across A, B, D genome of common wheat (*Triticum aestivum* L.)^18^, and H genome of barley (*Hordeum vulgar* L.)^19^ were developed using CHORUS2 v2.0.1^20^. BWA v0.7.17^21^ is utilized to align Hi-C data to the draft genome reference. Subsequently, contigs and Hi-C alignment were classified based on these homologous probes. Classified contigs were subjected to chromosome construction through the polyploid workflow of ALLHiC^22^. Juicebox v1.11.08^23^ was used to further manually correct the chromatin contact matrix and built the Hi-C interaction heatmap. SubPhase v1.2.6^24^ (kmer = 15) with default parameters was used to distinguish between two subgenomes of *E. sibiricus*. An H genome specific transposable element (Gypsy-96_TAe-LTR) was obtained by a pipeline procedure of RepeatExplorer^25,26^ using low coverage NGS sequencing data of both H genome donor species *Hordeum bogdanii* and St genome donor species *Pseudoroegneria stipifolia*. The content of the Gypsy-96_TAe-LTR was estimated hundreds times more in H genome than St genome. We used this element to further confirm which set of subgenomes is H and which set is St (Table 2). Benchmarking Universal Single-Copy Orthologs^27^ (BUSCO v5.2.2) and LTR Assembly Index^28^ (LAI) were employed to evaluate the completeness and contiguity of genome assemblies. Finally the assembly resulted in a genome size of 6.929 Gb with an contig N50 of  49.518 Mb (Table 3). Using SubPhaser and subgenome-specific repetitive sequence, we were able to successfully separate the two sets of subgenomes (Fig. 2).

**Table 2:** Alignment counts of the subgenome-specific repetitive sequence.

chromosome	SubGenome 1	SubGenome 2
chr1	4871	163
chr2	6345	468
chr3	6501	174
chr4	9820	198
chr5	4440	217
chr6	6482	162
chr7	5437	181

**Table 3:** Features of the *E. sibiricus* genome assembly and annotation.

Assembly characteristics	Values
Total length of contigs	6,929,461,306 bp
Total number of contigs	936
Contig N50	49,518,364 bp
Anchored rate of bases to the pseudochromosomes	99.37%
GC content	45.42%
Percentage of repetitive elements	82.49%
Complete BUSCOs	4857(99.2%)
Fragmented BUSCOs	2(0.0%)
Missing BUSCOs	36(0.8%)
Single base accuracy rate	99.98%
raw score of LTR Assembly Index	18.02
Score of LTR Assembly Index	12.61
Number of annotated genes	89,800

### Annotation of repetitive sequences and function gene

LTRfinder v1.07^29^ (-w 2 -C -D 15000 -d 1000 -L 7000 -l 100 -p 20 -M 0.85) and LTRHarvest v1.6.5^30^ (-minlenltr 100 -maxlenltr 7000 -mintsd 4 -maxtsd 6 -motif TGCA -motifmis 1 -similar 85 -vic 10 -seed 20 -seqids yes) were used to initially predict Long Terminal Repeat (LTR) sequences. Subsequently, LTR_retriever v2.9.5^31^ was used to merge the results and obtain the final LTR predictions. A De Novo repeat sequence database for *E. sibiricus* was constructed using RepeatModeler v2.0.3^32^ with default parameters. The final repeat sequence predictions were conducted using RepeatMasker v4.1.2^33^ pipeline.

The BRAKER3 v3.0.3^34^ pipeline was used for structural annotation of *E. sibiricus* genome. This comprehensive pipeline incorporated three sources of extrinsic evidence: short-read RNA-seq data obtained from the public NCBI Illumina dataset (SRP101478)^35^, full-length transcriptome sequencing from the current experiment, and protein sequences of Eukaryota sourced from OrthoDB^36^. BRAKER3 utilizes the GeneMark-ETP v1.02^37^ pipeline for gene prediction. This involves assembling transcript sequences with StringTie v2.2.1^38^. Short RNA-Seq reads were aligned to the genome by HISAT2 v2.2.1^39^. GeneMarkS-T analyzes the assembled transcripts to predict protein-coding genes, which are then searched against a protein database. ProtHint maps homologous proteins back to the genome, generating hints for another round of gene structure prediction. AUGUSTUS v3.4.0^40^ is trained on the high-confidence gene set and predicts a second genome-wide gene set with hint support. The predictions from these components were integrated using TSEBRA^41^.

**Fig. 3:**
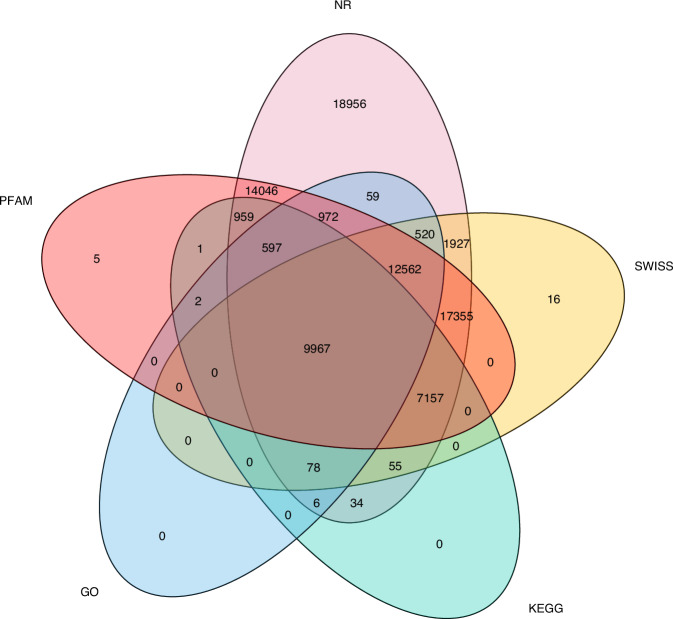
The venn picture of the function genes of *E. sibiricus* by using different database.

This study found that repetitive sequences accounted for 82.49% of the genome in *E. sibiricus* (Table 4). A total of 89,800 protein-coding genes were annotated, with an average gene length of 2,315 bp and an average CDS length of 1,075 bp (Table 5). Among these annotated genes, 85,250 genes were annotated in the NR^42^ database, 49,637 in the Swiss-Prot^43^ database, 63,623 in the Pfam^44^ database, 24,763 in the GO^45^ database, and 18,856 in the KEGG^46^ database. Additionally, 85,274 genes are annotated in at least one of these databases (Fig. 3).

**Table 4:** Classification of repeat annotation in *E. sibiricus*.

	Class	count	%masked
LTR	Copia	492,810	16.07%
	Gypsy	1,558,928	42.76%
DNA transposons	hobo-Activator	71139	0.30%
	Tc1-IS630-Pogo	163951	0.46%
	Tourist/Harbinger108608	108608	0.58%
Small RNA		29651	0.26%
Satellites		86231	1.10%
Simple repeats		451701	0.38%
Total			82.49%

**Table 5:** Statistics of the gene prediction.

Gene sets	Gene number	CDS region (M)	Average CDS length (bp)	Average exons per gene	Average exon length (bp)	Average intron in cds length (bp)
*E. sibiricus*-H	43482	52.45	1093	4.0	271	455
*E. sibiricus*-St	46290	54.17	1058	3.9	269	450
*E. sibiricus*	89800	106.65	1075	4.0	270	452

### Phylogenetic tree construction

**Fig. 4:**
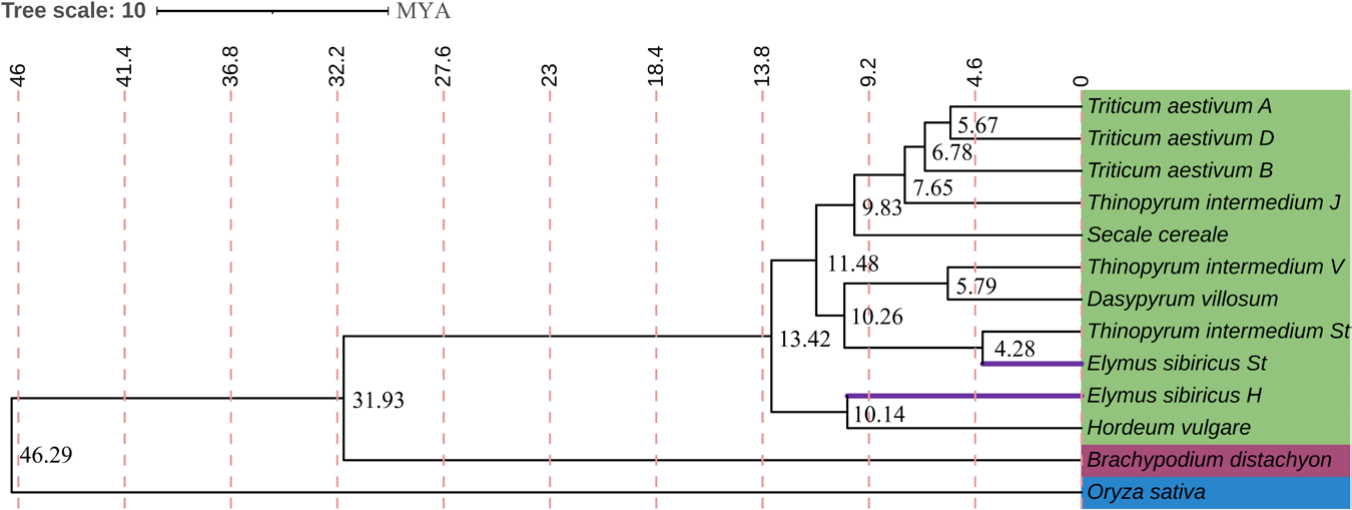
Estimation of divergence time between *E. sibiricus* and related species. Divergence times (unit: MYA) are indicated at each node. Green represent *Triticeae* species; red represent *Brachypodieae* species; blue represent *Oryzeae* species.

We have selected the Coding DNA Sequences(CDS) of the following genomes for phylogenetic analysis: *Oryza sativa*^47^, *Brachypodium distachyon*^48^, *Triticum aestivum* (subgenomes A, B, and D), *Secale cereale*^49^, *Thinopyrum intermedium* (subgenomes St, J^r^, and J^vs^) (https://phytozome-next.jgi.doe.gov/info/Tintermedium_v3_1), *Dasypyrum villosum*^50^, *Hordeum vulgare* along with *E. sibiricus* (subgenomes H and St). Orthofinder v2.5.5^51^ with the search engine Blast v2.14.1^52^ was employed to identify orthologous genes. From the selected genomes, a total of 2,082 lineal homologous genes were obtained. MUSCLE v5.1^53^ was used for multiple sequence alignment. The phylogenetic tree was constructed using RAxML v8.2.12^54^ with the maximum likelihood method. Divergence times were estimated with mcmctree v4.10.7^55^ using the calibrated times (*O. sativa* - *B. distachyon*: 41.5–62.0 MYA) from the Time Tree^56^ website (Fig. 4).

### synteny analysis

One Step MCScanX in TBtools-II^57^ was used for synteny analysis. First,coding protein sequences between subgenomes were aligned using blastp v2.15.0 + (−evalue 1e-5 -num_alignments 5), MCScanX v2022.11.01^58^ with default parameters was employed to identify collinear blocks.

## Data Records

The raw sequence data reported in this paper have been deposited in the Genome Sequence Archive in National Genomics Data Center^59^, China National Center for Bioinformation/Beijing Institute of Genomics^60^, Chinese Academy of Sciences (GSA: CRA014200)^61^. The final chromosome assembly of *E. sibiricus* was deposited at GenBank under the accession number JBDKXM000000000^62^. Genome assembly and annotation, conserved homologues probes and subgenome-specific repetitive sequnce were uploaded to figshare^63^.

## Technical Validation

**Fig. 5:**
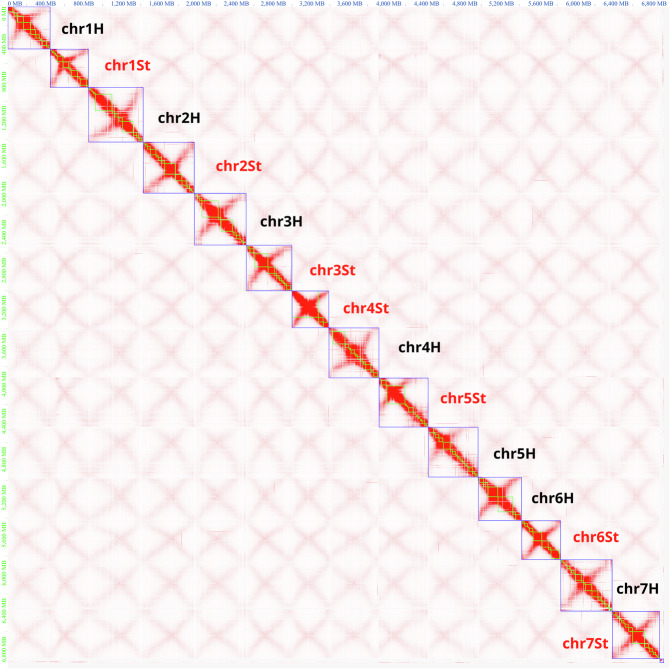
Contact map after the integration of the Hi-C data and manual correction. blue boxes represent pseudo molecules; green boxes represent contigs.

The genome-wide Hi-C interaction heatmap was generated using Juicerbox. The coordinates in the heatmap represent all bins on individual chromosomes, where the color of each point indicates the logarithmic value of the corresponding bin pair interaction strength in the genome (Fig. 5). The interaction strength intensifies from white to red, with darker colors indicating higher interaction strength. Notably, regions with higher interaction strength exhibit deeper colors, and the depth of colors along the diagonal is significantly higher than at the two ends. The anti-diagonals are typical for Triticeae genomes and correspond the Rabl configuration of Triticeae chromosomes^64,65^. Following manual adjustments, the current assembly of the *E. sibiricus* genome adheres to the distance-dependent interaction decay. From the global heatmap perspective, the overall assembly results appear satisfactory, with no apparent clustering errors between chromosomes.

The ultimate calculated LTR Assembly Index (LAI) value is 12.61, with a corresponding raw LAI of 18.02. In accordance with the criteria proposed by the authors of the LTR_retriever methodology, the assembly quality of the *E. sibiricus* is categorized at the reference level.

The BUSCO analysis of the entire genome indicates a high level of completeness and contiguity in the assembly of the *E. sibiricus* genome. Among the 4895 single-copy gene set, only 38 single-copy genes were found to be either missing or fragmented. We also conducted BUSCO analysis by extracting the longest transcript of each gene. The results indicate a relatively complete annotation, with the majority of genes on subgenomes being identified as single-copy (Table 6).

**Table 6:** BUSCO estimation for *E. sibiricus* genome assembly and annotation.

	Complete and single-copy	Complete and duplicated	Fragmented	Missing
*E. sibiricus-*H Genome	4533(92.59%)	233(4.76%)	33(0.67%)	97(1.98%)
*E. sibiricus*-St Genome	4464(91.18%)	305(6.23%)	26(0.53%)	101(2.06%)
*E. sibiricus* Genome	464(9.48%)	4394(89.75%)	4(0.08%)	34(0.69%)
*E. sibiricus-*H Gene	4413(90.13%)	190(3.88%)	33(0.67%)	260(5.31%)
*E. sibiricus-St* Gene	4325(88.34%)	249(5.09%)	31(0.63%)	291(5.94%)
*E. sibiricus* Gene	629(12.85%)	4219(86.17%)	4(0.08%)	44(0.90%)

Phylogenetic analysis with the assembled CDS showed close relationships between St genome in *E. sibiricus* and St in *Th. Intermidum*, and those between H genome in *E. sibiricus* and *H. vulgare*, which is accordant with the recognized genome constitution of *E. sibiricus*.

The synteny analysis revealed an apparent collinearity distort in 4H and 6H chromosome (Fig. 1), which was confirmed by a species-specific 4H/6H reciprocal translocation detected by chromosomal Florescence *in situ* hybridization with single-gene probes in *E. sibiricus*^8^.

## Data Availability

All software and pipelines were executed according to the manual and protocols of the published bioinformatics tools. The version and parameters of software have been described in Methods.
